# Supplementary material for: Nasal Tumor Vaccination Protects against Lung Tumor Development by Induction of Resident Effector and Memory Anti-Tumor Immune Responses
Source: Pharmaceutics. 2023 Jan 29;15(2):445. doi: 10.3390/pharmaceutics15020445 (PMC9958580; doi:10.3390/pharmaceutics15020445)
Supplement: Supplementary file 1 [file pharmaceutics-15-00445-s001.zip › pharmaceutics-2176173-supplementary.pdf]

pharmaceutics

MDPI

Supplementary Materials: Nasal Tumor Vaccination Protects against Lung Tumor Development by Induction of Resident Effector and Memory Anti-tumor Immune Responses

Michael Donkor, Jamie Choe, Danielle Marie Reid, Byron Quinn, Mark Pulse, Amalendu Ranjan, Pankaj Chaudhary and Harlan P. Jones

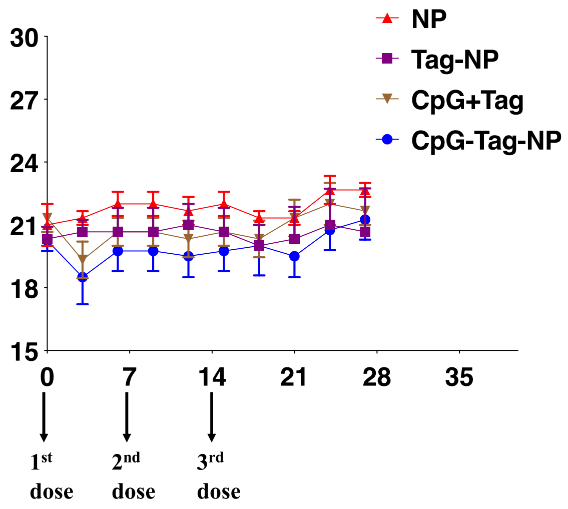

Figure S1. Effect of Intranasal CpG-NP-Tag immunization on weight of mice. Intranasal vaccine dosing regimen and its effect on mice weights.

A. CD8+ T cells.

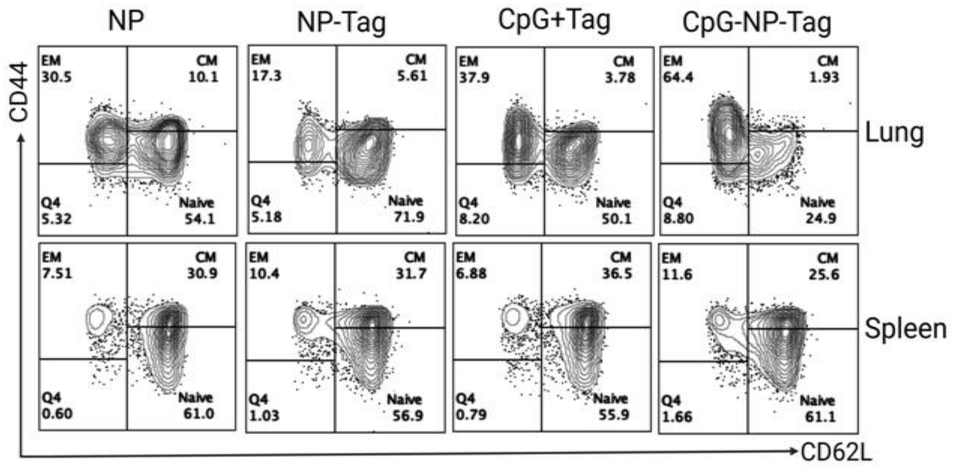

B. CD4+ T cells.

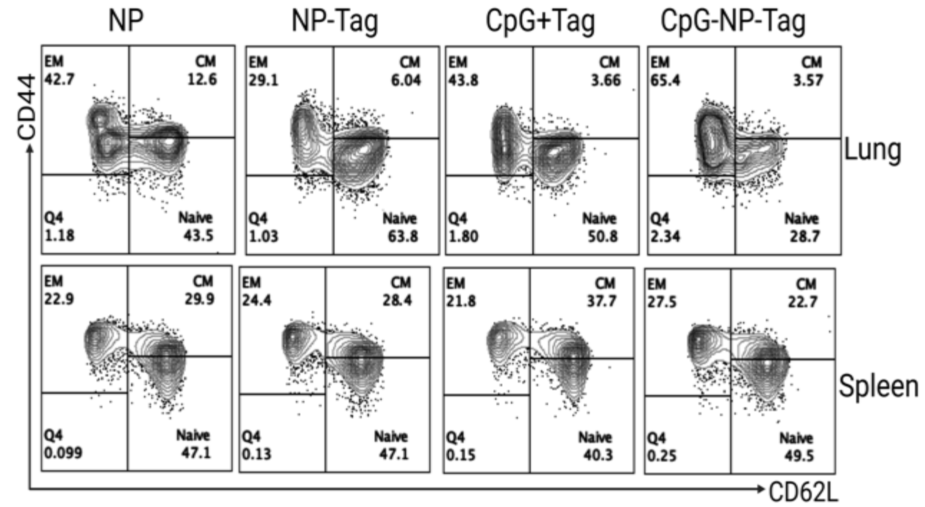

Figure S2. Intranasal CpG-NP-Tag immunization increases accumulation of effector/effector memory T-cell at the expense of naïve T-cells in the lungs of Balb/c mice (A and B) Representative FACS blot of CD44 and CD62L expression among CD8+ (A) and CD4+ (B) T cells from lungs and spleen of experimental mice day 7 post immunization.

A CD8+ T cells.

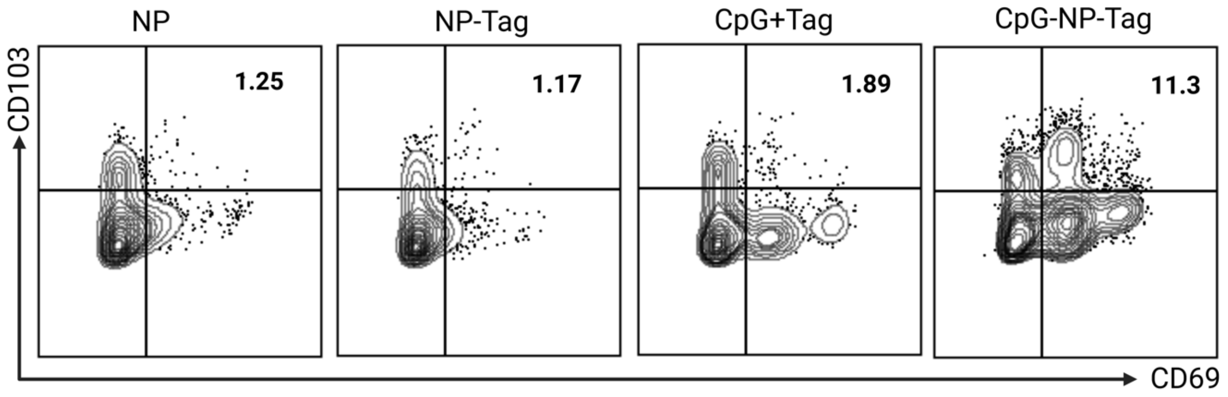

B CD4+ T cells.

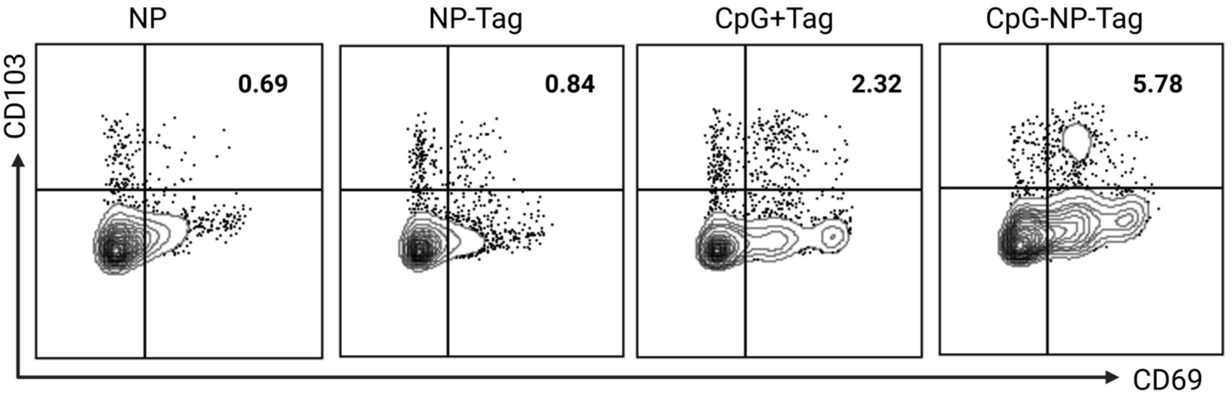

Figure S3. Intranasal CpG-NP-Tag immunization increases accumulation of lung resident memory T-cells in the lungs of Balb/c mice (A and B) Representative FACS blot of CD103 and CD69 expression among $CD8^+$ (A) and $CD4^+$ (B) T cells from lungs of experimental mice day 7 post immunization.
